# The nymph and imago of Chinese mayfly *Siphlonurus davidi* (Navás, 1932)

**DOI:** 10.3897/zookeys.607.9159

**Published:** 2016-07-26

**Authors:** Yi-Ke Han, Wei Zhang, Ze Hu, Chang-Fa Zhou

**Affiliations:** 1 The Key Laboratory of Jiangsu Biodiversity and Biotechnology, College of Life Sciences, Nanjing Normal University, Nanjing 210046, China

**Keywords:** China, evolution, mayfly, *Siphlonurus*, wing

## Abstract

The imagos and nymphs of *Siphlonurus davidi* (Navás, 1932) are described for the first time. The adult has colourful wings and cross veins, the MP is forked asymmetrically at its base, a long cubital area is present with more intercalaries, and it has a relatively simpler penis and larger hindwings compared to its congeners. The venation and genitalia show that it is a plesiomorphic species in the genus. A key to the Asian species of *Siphlonurus* with coloured wings is provided in conclusion.

## Introduction

The species *Siphlonurus davidi* (Navás, 1932), which was based on a single male subimago, was re-described by Sartori and Peters in 2004. The damaged type consists of a twisted sub-imaginal specimen, which shows unclear characteristics and makes its systematic position questionable. On the other hand, because of the long cubital area of its forewing, the relatively broader hindwing, and the simple genitalia, it shows some valuable phylogenetic characters. Using these, Navás (1932) originally placed it in the genus *Siphluriscus* Ulmer, 1920, which is currently considered to possess the highest number of plesiomorphies in the order Ephemeroptera (Zhou and Peters 2003; Ogden et al. 2009). However, the poor state of the sub-imaginal type precludes deeper investigations and discussion.

In 2013, a Chinese professor working on aquatic insects collected within a national park in Sichuan province (southwestern China), the same province where the type of *Siphlonurus davidi* was originally found. Among his mayfly collection, some *Siphlonurus* nymphs and imagos were present: the imago wings had distinct pigmented spots and markings. After careful examination and comparison with the good quality photographs in Sartori and Peters (2004), they were recognized as *Siphlonurus davidi*. These specimens will greatly increase our knowledge and understanding of this species, so they are described and illustrated here. Furthermore, venation and male genitalia of imagos show it is a valid species which has some plesiomorphies.

## Materials and methods

1♂2♂♂subimagos, 4♀♀subimagos and 25 nymphs, Jing Hai (Mirror pool or lake, alt. 2398m), 2013-VII-6; 2♀♀5 nymphs, Xi-Niu Hai (rhinoceros pool, alt. 2348 m), 2013-VII-7, leg. Beixing Wang; 1♀ and 36 nymphs, Lao-Hu Hai (tiger pool, alt. 2439 m), 2013-VII-7, leg. Hun He and Guangba Li; 50 nymphs, Jia-zu Hai (bamboo pool, alt. 2744 m), 2013-VII-6, leg. Yong Cao.

All specimens were collected at Jiu-Zhai-Guo (Jiuzhai Valley), Sichuan Province, China, and now are deposited in Mayfly Collection, College of Life Sciences, Nanjing Normal University, China. The nymphs were sampled from pools or lakes and imaginal materials were attracted to and collected by lights.

## Results

### Siphlonurus davidi

Taxon classificationAnimaliaEphemeropteraSiphlonuridae

(Navás, 1932)

Siphluriscus davidi Navás, 1932: 929, fig. 46, male subimago. Type: male subimago, from China (Sichuan=Se-Tchouen).
Siphluriscus davidi [sic.]: Ulmer, 1936: 215.
Siphluriscus davidi : Wu, 1935: 251; Gui, 1985: 80.
Siphlonurus davidi : Zhou & Peters, 2003: 346 (tentatively); Sartori & Peters, 2004: 2, figs 1–7 (redescription on type and transfer).

#### Distribution.

China (Sichuan).

#### Description.

**Figure 1.**
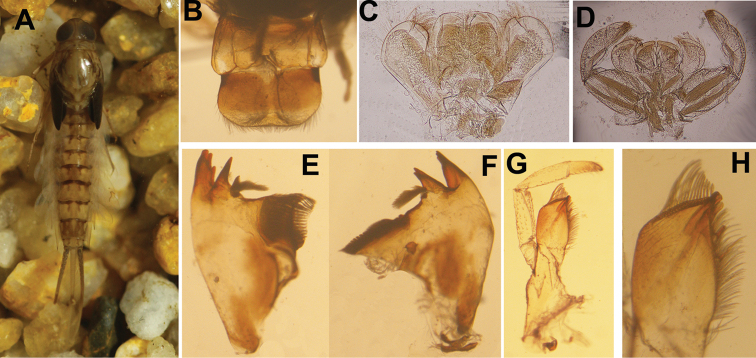
Nymphal characters of *Siphlonurus davidi*. **A** habitus **B** labrum and clypeus **C** hypopharynx **D** labium **E** right mandible **F** left mandible **G** maxilla **H** apex of maxillary lacia-genicia

**Figure 2.**
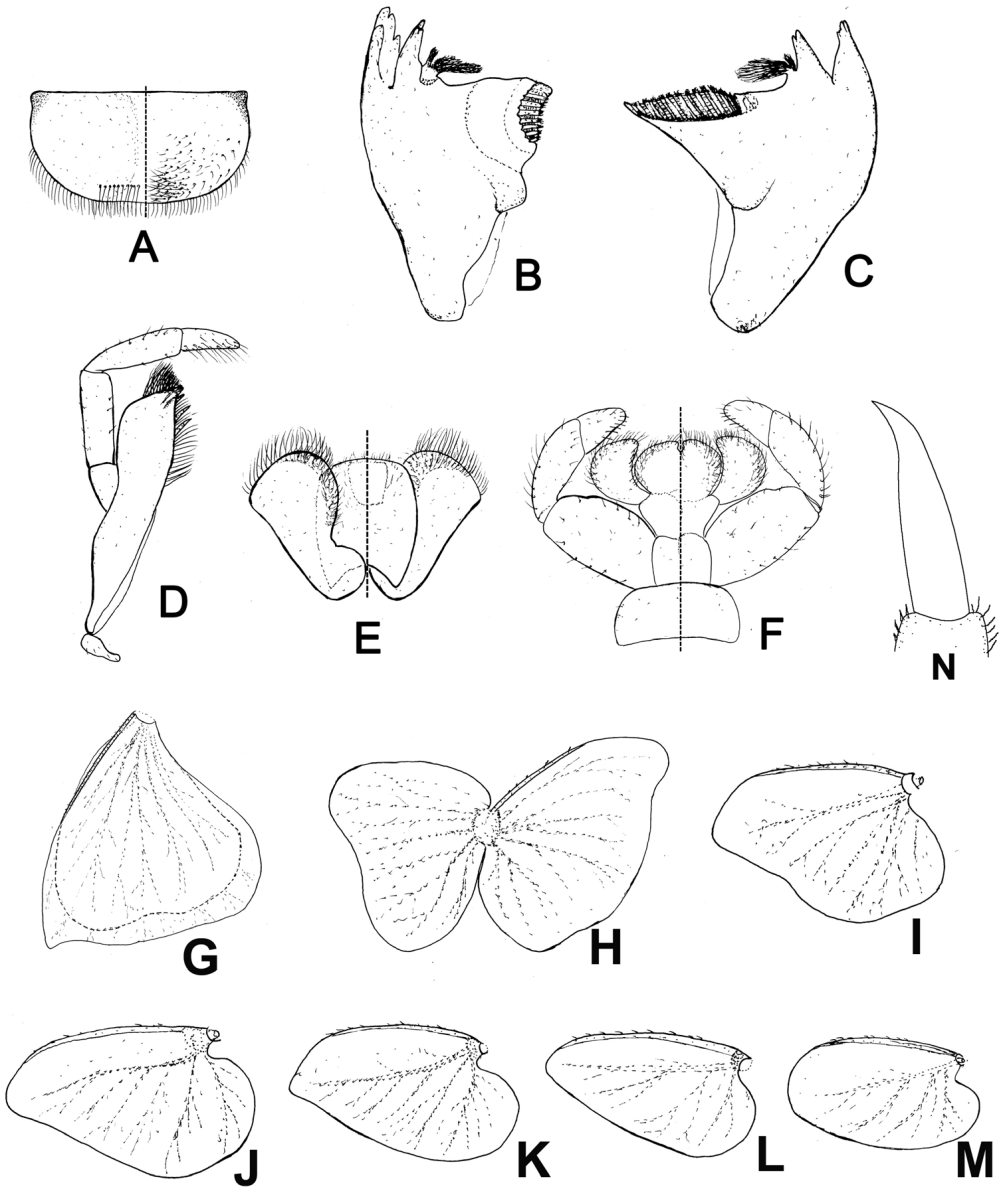
Mouthparts and gills of *Siphlonurus davidi* nymph. **A** labrum **B** right mandible **C** left mandible **D** maxilla **E** hypopharynx (dorsal view on left; ventral view on right) **F** labium (dorsal view on left; ventral view on right) **G**–**M** gills 1-7 (double lamellae of gills separated) **N** claw

**NYMPH** (in alcohol, Figs 1–2).

*Body length* 15.0–20.0 mm, caudal filament 6.0–7.0 mm, yellowish brown; head mostly obscured by compound eyes, hypognathous, length of antenna subequal to width of head, surface of antennae with very sparse tiny setae (Fig. 1A); *Mouthparts*: clypeus extended; labrum with obvious median groove, free margin with setae, an additional row of setae on dorsal surface near anterior margin; ventral surface with shorter setae; posterolateral corner slightly sclerotized (Figs 1B, 2A). Outer incisor of left *mandible* apically divided into three teeth, inner incisor with two teeth, prostheca constituted by two tufts of spines with common stem (Figs 1F, 2C); apex of right outer mandibular incisor serrated into four teeth, inner one with three teeth, prostheca also divided into two groups of numerous spines (Figs 1E, 2B); galea-lacinea of *maxilla* with a row of spines on crown, apex of maxilla divided into three broad denticles (maxillary canines *sensu* Kluge, 2004), upper half of inner margin with two rows of spines, three of them broader than others (dentisetae *sensu* Kluge, 2004), lower half with a row of setae (Figs 1G,H, 2D); *maxillary palpi* 3-segmented, basal one and second one subequal in length, apical one about 0.6× length of second one, surface of all segments with sparse setae, those on apical one slightly longer (Figs 1G, 2D). *Hypopharynx* (Figs 1C, 2E): lingua sub-quadrate, apical margin with short setae; superlinguae with longer setae on apical margin and lateral area. *Labium* with heart-shaped, unfused glossae and paraglossae, the latter slightly narrower but longer than the former; aboral surface with long hair; labial palpi 3-segmented, progressively shorter from base to apex, surface with setae and spines, those on apical segment longer (Figs 1D, 2F).

*Thorax*: all legs similar, femora with broad median marking bands, tibiae pale, tarsus with basal and apical colour rings, the latter one darker; length of femora: tibiae: tarsus ca. 1.8: 1.0: 1.5, surface with very short sparse spines and setae; mid- and hind-legs with clear patellar-tibial suture. *Claws* relatively slim and simple, without teeth (Fig. 2N).

*Abdomen*: Each tergite with three pairs of stripes dorsally; one pair parallel near median line, one at lateral margin, one oblique pair between them. Colour of tergites 3, 6, 9 slight darker than others; each tergite with a pair of short median stripes. Posterolateral corner of each tergite extended into sharp spines, progressively larger and wider from anterior to posterior (Fig. 1A). *Gills* on abdominal segments 1–7; gills 1–2 similar in shape and structure, with two lamellae, dorsal one slightly broader than ventral one, the former with sclerotized leading marginal line while the latter with a emarginated outer margin (Fig. 2G, H); gills 3–7 single, progressively shorter from anterior to posterior, tracheae gray and well visible; leading margin of gills 3–7 sclerotized, with small spines (Fig. 2I–M); cerci with long setae on mesal margin and tiny spines on articulations, terminal filament with long hair on both sides and spines between segments (Fig. 1A).

**Figure 3.**
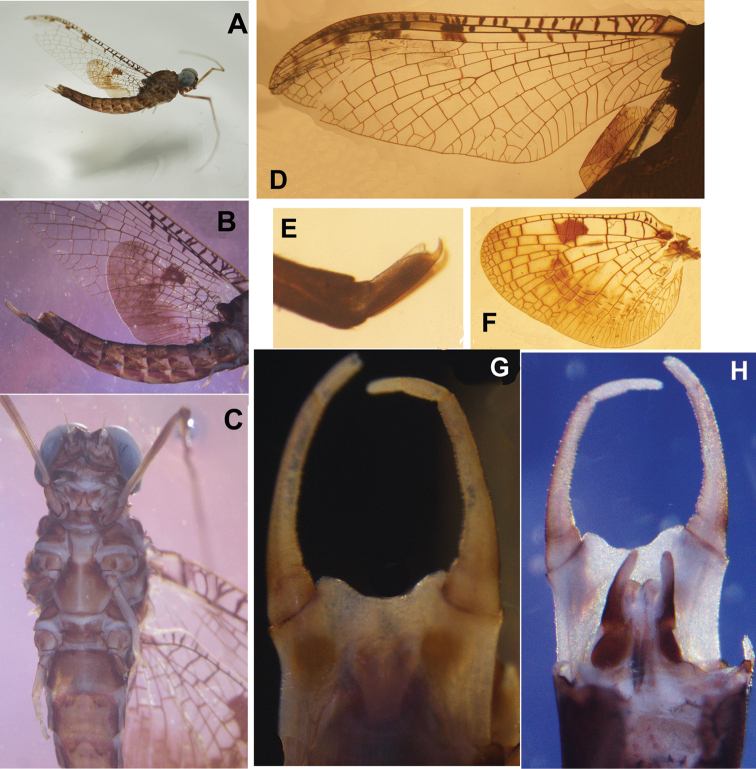
Male structures of *Siphlonurus davidi* **A** habitus **B** abdomen (lateral view) **C** front body (ventral view) **D** forewing **E** claws **F** hindwing **G** genitalia (ventral view) **H** genitalia in dorsal view

**Figure 4.**
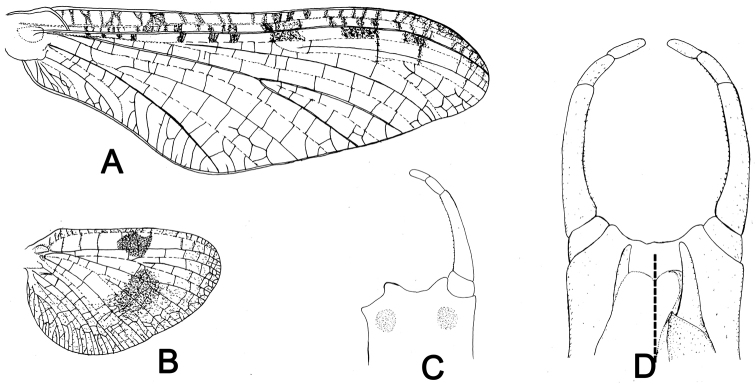
Male structures of *Siphlonurus davidi* **A** forewing **B** hindwing **C** genitalia (ventral view) **D** genitalia in dorsal view (dorsal view of penes on right; ventral view on left).

**MALE** (in alcohol, Figs 3–4).

General colouration reddish brown, with pale sutures and grooves on body (Fig. 3A–C); body length 13.0 mm, forewing 13.0 mm, hindwing 6.0 mm, antennae 2.0 mm. *Head*: compound eyes widely contiguous, each of them spherical, upper portion grey, lower portion black, a clear line between them (Fig. 3A, C). *Thorax*: coxa and trochanter of foreleg deeply pigmented with reddish brown in colour, and apical half of femora, tibia and tarsus also brown but basal half of each is pale (Fig. 3A); length ratio of femora, tibia and tarsus of foreleg = 2: 1: 3.7, five segments of fore-tarsus progressively shorter from distally; two claws similar, acute, hooked (Fig. 3E); mid- and hind-legs apparently vestigial in the single male imago (this may be due to damaged or broken legs in the previous life stage of this specimen) but normal in females and male sub-imagos (Fig. 3A, C). *Wings*: base of forewing slightly pigmented, cross veins between C, Sc, R_1_ and R_2_ surrounded with distinct pigments (Figs 3A–D, 4A); MA and Rs with long common stem, further jointing with MP, then stem of them fused with R_1_ or run along it. MA fork distal to middle of wing, MP fork at very base, just slightly more distal than fork of Rs and MA, MP_2_ strongly bent backwards at base, very close to CuA, thus making the MP area relatively broad; CuA slightly curved backwards, joining margin of forewing just before tornus; 6-9 relatively longer attaching veins between CuA and posterior margin, 1-3 may be fork further; CuP stemmed with CuA clearly at base, curved strongly backwards, slightly longer than half of CuA; A_1_ attached posterior margin with two veinlets (Figs 3D, 4A). Base and cross veins of hindwing clearly pigmented, especially those between C and Sc veins; an additional large dark patch at middle of Sc and R_1_ cells; outer half of hindwing washed with reddish colour, it makes this area semi-transparent, area near centre of hindwing darker than others; MA fork at distal 1/3 point, Rs fork more basal than MA, MP fork basal to middle of hindwing (Figs 3F, 4B); ratio of width: length about 0.65. *Abdomen*: each tergite with a pair of brown stripes in middle, another pair of longer oblique stripes near anterolateral corner, lateral margin of terga strongly and broadly pigmented (Fig. 3A–B); each sternite with a pair of indistinct short median marks, anterolateral corner and lateral margins clearly pigmented (Fig. 3C). *Genitalia*: subgenital plate deeply emarginated, ventral surface with two large round brown marks (Figs 3G, 4C); forceps 4-segmented, basal one shortest but broadest, second segment about twice length of third and apical segments together, the latter two subequal in length, each slightly longer than basal one, inner margin of forceps with tiny projections; penes short, invisible in ventral view, basal half of penis broad, with a large broad membranous lobe in ventral; apical half slim (Figs 3H, 4D). Cerci lost, terminal filament vestigial.

**Figure 5.**
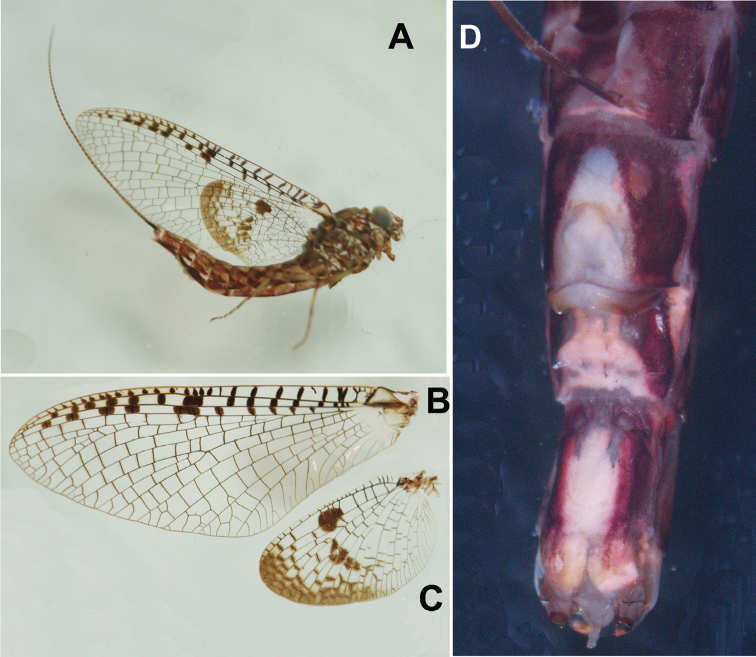
Female structures of *Siphlonurus davidi*. **A** habitus **B** forewing **C** hindwing **D** posterior part of abdomen (ventral view).

**FEMALE** (in alcohol, Fig. 5).

Body length 12.0–13.0 mm, forewing 12.0–13.0 mm, hindwing 6.0–7.0 mm, cerci 15 mm; body colour pattern similar to male imago (Fig. 5A). Two compound eyes separated widely, distance between them near to width of eye. Ratios of foreleg femora: tibiae: tarsus lengths = 2.5: 1.8: 3.0, that of midleg and hindleg = 2.5: 1.5: 2.2; tarsus 5-segmented but basal one fused with tibiae partially, fourth segment shortest, others progressively shorter from basal to apical; two claws of all legs with hooked apex. *Forewing*: all cross veins surrounded with darker pigments than male, especially those at outer half portion (Fig. 5A–B). *Hindwing*: base pigmented, all cross veins covered with distinct colour, distal half darker, two additional dark patches near middle (Fig. 5A, C); posterior margin of sternite 7 thickened and extended slightly (Fig. 5D). Ceri reddish brown, base and articulations darker; terminal filament tiny, pale.

**Figure 6.**
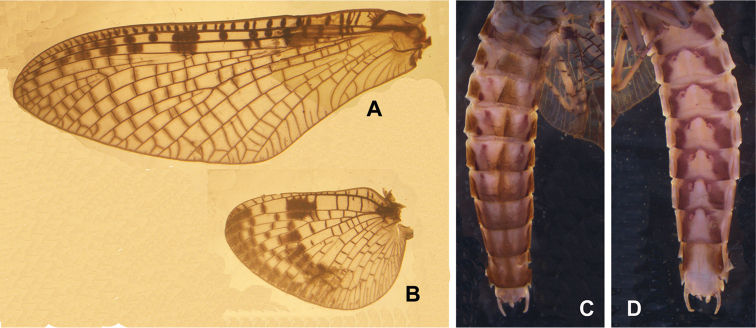
Male subimaginal structures of *Siphlonurus davidi*. **A** forewing **B** hindwing **C** abdomen (dorsal view) **D** abdomen (ventral view).

**MALE SUBIMAGO** (in alcohol, Fig. 6).

Similar to male but duller. *Femora*: ratio of tibiae: tarsus of foreleg = 1.0: 0.6: 1.2, that of mid- and hind-legs 1.0: 0.6: 0.9. Colour pattern of abdominal terga and sterna similar to male but clearer (Fig. 6C–D). Subgenital plate only shallowly curved, posterior margin waved.

**FEMALE SUBIMAGO** (in alcohol).

Similar to female imago in colour pattern but opaque. Ratio of femora: tibiae: tarsus of legs = 1.0: 0.6: 1.0.

**EGGS** (Fig. 7).

Generally oval but one pole larger than the other, approximately 150 µm in length and 100 µm in width, without polar cap. Exochorionic surface uniform, consisting of irregular ridges or rims.

**Figure 7.**
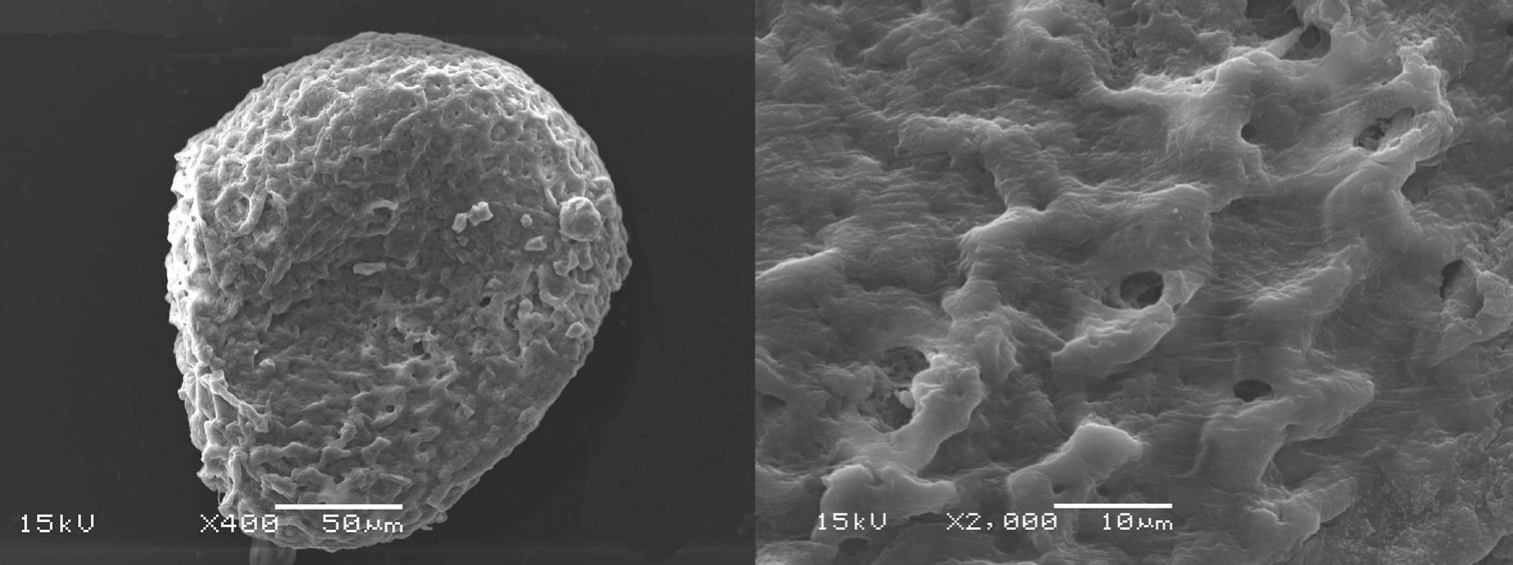
Egg of *Siphlonurus davidi* (SEM image). **A** shape and surface of Egg **B** surface enlarged.

##### Key to three Asian *Siphlonurus* species with coloured wings (male)

**Table:** 

1	Ventral lobe of penes with teeth	**2**
–	Ventral lobe of penes without teeth	***Siphlonurus davidi***
2	All cross veins of forewing pigmented, fore- and hindwings with colourful stripes and spots	***Siphlonurus palaearcticus***
–	Cross veins of wings without pigment, hindwing may with central spot but without stripe	***Siphlonurus binotatus***

##### Key to three Asian *Siphlonurus* species with coloured wings (female)

**Table:** 

1	All cross veins of forewing pigmented, fore- and hindwings with colourful stripes and spots	**2**
–	Cross veins of wings without pigment, hindwing may with central spot but without stripe	***Siphlonurus binotatus***
2	Cross veins between C to Rs of forewing clearly surrounded with colourful pigments; distal half of hindwing with colour, semi-transparent	***Siphlonurus davidi***
–	Cross veins of whole forewing surrounded with pigments; hindwings wing pigments surrounding cross veins, other parts transparent	***Siphlonurus palaearcticus***

##### Key to three Asian *Siphlonurus* species with coloured wings (mature nymph)

**Table:** 

1	Abdomen with clear, obvious, relatively wide trachea or thread-like markings	***Siphlonurus binotatus***
–	Abdomen maybe with various markings but without distinct above colour pattern	**2**
2	Obvious posterolateral spines present on terga 1–9	***Siphlonurus davidi***
–	Obvious posterolateral spines present on terga 3–9	***Siphlonurus palaearcticus***

##### Remarks

Approximately 40 *Siphlonurus* species have been reported from the Nearctic and Palaearctic realms, Eurasia hosting half of them (Kluge, 2004). Just as Sartori and Peters (2004) pointed out, *Siphlonurus davidi* is close to the *Siphlonurus palaearcticus* (Tshernova, 1930) and *Siphlonurus binotatus* (Eaton, 1892) because all of these three species have colourful wings in imagos. However, the imagos of *Siphlonurus davidi* can be differentiated from the latter two by the following characters:

1) The forewing of *Siphlonurus davidi* has more pigmented patches than that of *Siphlonurus binotatus* but fewer than *Siphlonurus palaearcticus*. According to Uéno (1928) and Gose (1979), *Siphlonurus binotatus* has only one conspicuous marking on forewings. On the contrary, *Siphlonurus palaearcticus* has numerous markings and spots on forewing, and a distinct dark stripe at middle. The forewings of *Siphlonurus davidi* have no stripe but spots and markings between C and R_2_ veins.

2) Compared to *Siphlonurus binotatus* and *Siphlonurus palaearcticus*, the MP on forewing of *Siphlonurus davidi* forks more basally, CuP more curved and cubital area is longer.

3) Unlike *Siphlonurus binotatus* and *Siphlonurus palaearcticus*, the hindwings of *Siphlonurus davidi* are more colourful. They have two obvious dark patches and half of the hindwing is pigmented and semi-transparent. On the contrary, *Siphlonurus binotatus* has only one clear stripe or patch near the centre, the other part of hindwing has no colour and is totally hyaline. The cross veins in the hindwing of *Siphlonurus palaearcticus* are pigmented but the patches are separated.

4) The penis of *Siphlonurus davidi* has only ventral membranous lobe, but the lobe of *Siphlonurus binotatus* and *Siphlonurus palaearcticus* have teeth on its apex.

In nymphs, the terga of *Siphlonurus davidi* and *Siphlonurus binotatus* have three pairs of stripes while that of *Siphlonurus palaearcticus* has only one pair. Similarly, all abdominal terga of *Siphlonurus davidi* and *Siphlonurus binotatus* nymph have distinct posterolateral spines, while the spines of *Siphlonurus palaearcticus* are only on segments 3–9 and much smaller. *Siphlonurus binotatus*, on the other hand, has obvious tracheae-like markings on the abdomen and obvious dark spots near the lateral margins of terga, which are not found in *Siphlonurus davidi* or *Siphlonurus palaearcticus*. The latter two species have different colour patterns on nymphal legs. The median half of femora of *Siphlonurus davidi* is washed with brown pigments, but that of *Siphlonurus palaearcticus* is paler. Both species have two brown rings on the tarsal base and apex respectively, while the apical one of *Siphlonurus davidi* is much darker than that of *Siphlonurus palaearcticus* The gill figures provided by Kluge (1982) and Uéno (1928, 1931) show the nymphal gills 2–7 of *Siphlonurus binotatus* and *Siphlonurus palaearcticus* have sclerotized leading margins, but all gills of *Siphlonurus davidi* nymph have these lines.

##### Plesiomorphic and autapomorphic characters

Based on the double gills 1–2, coxae and mouthparts without gills, simple claws of nymphs and colourful wings, the distal fork MA of hindwing, the fused subgenital plate, and complex penes of imagos, *Siphlonurus davidi* is definitely a species which belongs in the Siphlonuridae. However, at least three characteristics show it is older than other species in the genus *Siphlonurus*. The first one is the forking point of MP which is sub-equal to that of fusion point of MA and Rs. In other *Siphlonurus* species, as far as we know, like in *Siphlonurus palaearcticus*, this point is more distal. The second character is the cubital area which is longer and with more intercalaries between CuA and the posterior margin of wing. The third structure mentioned here is the hindwings of *Siphlonurus davidi*, which are approximately half the length of forewings, longer than in other *Siphlonurus* species (less than half). It should be pointed out that these three characters of *Siphlonurus davidi* are also found in *Siphluruscus chinensis* (Siphluriscidae), which is clearly a basal clade of Ephemeroptera; therefore, these characters are considered as plesiomorphic.

The MP vein in forewing of *Siphlonurus davidi* is somewhat unique. It forks asymmetrically at the base, then MP_2_ bends backwards strongly near to CuA. This condition is common in Ephemeridae and Potamanthidae, and is similar to *Siphluruscus chinensis*, but it seems that it is not found in other siphlonurids.

## Supplementary Material

XML Treatment for Siphlonurus davidi
